# The complete chloroplast genome sequence of *Elaeocarpus decipiens* (Elaeocarpaceae)

**DOI:** 10.1080/23802359.2021.1984333

**Published:** 2021-10-05

**Authors:** Guangbo Zhang, Zhen Wang, Tengfei Yan

**Affiliations:** Xinyang Agriculture and Forestry University, Xinyang, Henan, China

**Keywords:** *Elaeocarpus decipiens*, plastid genome, phylogeny, Elaeocarpaceae

## Abstract

*Elaeocarpus decipiens* F. B. Forbes and Hemsl is an evergreen tree native to East Asia. Here, we sequenced and reported the complete chloroplast genome of *E. decipiens* for the first time. In the present work, the complete chloroplast (cp) genome sequence of *E. decipiens* was characterized by Illumina pair-end sequencing. The genome was 158,148 bp in total length, including a large single-copy (LSC) region of 85,702 bp, a small single-copy (SSC) region of 17,672 bp, and a pair of invert repeats (IR) regions of 27,387 bp. The plastid genome contained 142 genes including 97 protein-coding genes, 37 tRNA genes, and eight rRNA genes. Phylogenetic analysis based on 13 chloroplast genomes indicates that *E. decipiens* is closely related to *Elaeocarpus bracean* in Elaeocarpacea.

*Elaeocarpus decipiens* F. B. Forbes and Hemsl (http://powo.science.kew.org) is a broad-leaved evergreen large shrub tree native to temperate East Asia, typically found in evergreen forests from 1300 to nearly 8000 feet in elevation through much of China and in Vietnam. It typically grows from 20 to 35 feet in height with an equal spread. Its stylish branching pattern, opulent growth, solid form, and beautiful tropical foliage make this plant species an attractive landscape tree (Moore et al. 2020). The chloroplasts (cp) genome has a maternal inheritance and conserved structure, that has been used to examine the developmental and phylogenetic relationships of plants (Wang et al. 2018). For this reason, we first assembled the complete cp genome of *E. decipiens* based on Illumina pair-end sequencing data, to understand the phylogenetic position of the species for future evolutionary studies.

*E. decipiens* leaves specimens were taken from Henan Province, China (Xinyang Agriculture and Forestry University: 114°13′E, 32°17′N) and quickly dried with silica gel for DNA extraction. DNA was sent to Wuhan Bena Biotechnology Co., Ltd. to construct a DNA library and sequenced by Illumina NovaSeq 6000 sequencing platform (Illumina, San Diego, CA, USA). Fresh leaves of the individuals were collected and flash-frozen in liquid nitrogen and then stored in a refrigerator (–80 °C) until DNA extraction. The Specimens have been stored in the Herbarium of the College of Horticulture, Plant Biotechnology Laboratory, Xinyang Agriculture and Forestry University (Resource person: Guangbo Zhang and Email: zgb1688@163.com) the specimen code ED. In addition, the Illumina High-throughput sequencing platform data were filtered by the script in the NOVOPlasty (Dierckxsens et al. 2017). After the DNA extraction from fresh leaf tissues, its quantification was validated using Agarose gel electrophoresis and Nanodrop concentration 500 bp randomly interrupted by the Covaris ultrasonic breaker for library construction. Approximately, 14.2 GB of raw data were generated with 150 bp paired-end read lengths. The complete plastid genome of *Elaeocarpus braceanus* (GeneBank accession: NC_054266) as a reference and plastid genome of *E. decipiens* both were assembled by GetOrganelle pipe-line (https://github.com/Kinggerm/GetOrganelle), it can get the plastid-like reads, and the reads were viewed and edited by Bandage (Wick et al. 2015). The cp genome annotation based on the comparison was assembled by Geneious v 11.1.5 (Biomatters Ltd., Auckland, New Zealand) (Kearse et al. 2012).

The final complete cp genome sequence of *E. decipiens* has been submitted to GenBank under the accession number; MZ334669. Raw reads were deposited in the GenBank Sequence Read Archive (SRA PRJNA731248). The complete cp genome of *E. decipiens* was a circular shape of 158,148 bp in length, consisting of four distinct regions: a large single-copy (LSC), a small single-copy (SSC), and a pair of inverted repeats (IR) with regions of 85,702 bp, 17,672 bp, and 27,387 bp, respectively. The complete cp genome consisted of 142 genes, including 97 protein-coding genes, 37 tRNA genes, and 8 rRNA genes. The complete cp genome GC content was 34.32%. Phylogenetic analyses including *E. decipiens*, *E. bracean*, *Elaeocarpus japonicus*, *Sloanea sinensis*, seven Oxalidaceae species, two Brunelliaceae species, and one Cunoniaceae species, were performed to use complete cp genomes. All of them were downloaded from NCBI GenBank. The sequences were aligned by MAFFT v7.307 (Katoh and Standley 2013), and the phylogenetic tree was constructed by RAxML (Stamatakis 2014). Both the *E. decipiens* and *E. bracean*, belonging to the Elaeocarpacea family, had a close relationship. The analysis of the cp genome of *E. decipiens* provides excellent genetic information for further studies of this precious species and the taxonomy, phylogenetics, and evolution of Elaeocarpacea (Figure 1).

**Figure 1.**
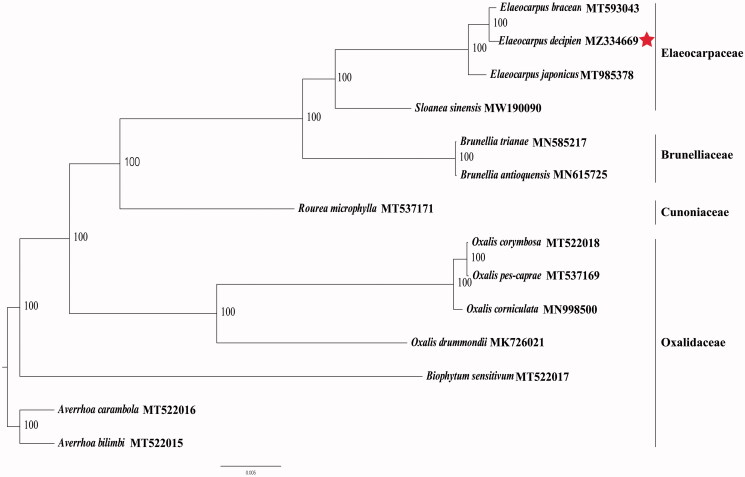
Maximum-likelihood phylogenetic tree based on complete cp genomes. Numbers close to each node are bootstrap support values.

## Data Availability

The data that support the findings of this study are openly available in GeneBank at https://www.ncbi.nlm.nih.gov/. The complete chloroplast genome generated for this study has been deposited in GeneBank with accession number MZ334669. The associated BioProject, SRA, and Bio-Sample numbers are PRJNA731248, SRX10944172, and SAMN19276134, respectively.
